# Diversity, distribution, and functional potentials of magroviruses from marine and brackish waters

**DOI:** 10.3389/fmicb.2023.1151034

**Published:** 2023-04-21

**Authors:** Bu Xu, Lu Fan, Wenxiu Wang, Yuanqing Zhu, Chuanlun Zhang

**Affiliations:** ^1^School of Environment, Harbin Institute of Technology, Harbin, China; ^2^Shenzhen Key Laboratory of Marine Archaea Geo-Omics, Department of Ocean Science and Engineering, Southern University of Science and Technology, Shenzhen, China; ^3^Southern Marine Science and Engineering Guangdong Laboratory (Guangzhou), Guangzhou, China; ^4^Shanghai Sheshan National Geophysical Observatory, Shanghai, China

**Keywords:** magroviruses, archaeal viruses, marine archaea, marine group II archaea, brackish water system

## Abstract

Marine group II (MGII) archaea (*Ca.* Poseidoniales) are among the most abundant microbes in global oceanic surface waters and play an important role in driving marine biogeochemical cycles. Magroviruses – the viruses of MGII archaea have been recently found to occur ubiquitously in surface ocean. However, their diversity, distribution, and potential ecological functions in coastal zones especially brackish waters are unknown. Here we obtained 234 non-redundant magroviral genomes from brackish surface waters by using homology searches for viral signature proteins highlighting the uncovered vast diversity of this novel viral group. Phylogenetic analysis based on these brackish magroviruses along with previously reported marine ones identified six taxonomic groups with close evolutionary connection to both haloviruses and the viruses of Marine Group I archaea. Magroviruses were present abundantly both in brackish and open ocean samples with some showing habitat specification and others having broad spectrums of distribution between different habitats. Genome annotation suggests they may be involved in regulating multiple metabolic pathways of MGII archaea. Our results uncover the previously overlooked diversity and ecological potentials of a major archaeal virial group in global ocean and brackish waters and shed light on the cryptic evolutionary history of archaeal viruses.

## Introduction

Viruses play an essential role in biogeochemical cycles of marine ecosystems by manipulating biomass production and population structure, by regulating host metabolism, and by evolving together with their microbial hosts (Zimmerman et al., 2020). Archaeal viruses are found to be much more diverse than bacteriophages (Krupovic et al., 2018; Baquero et al., 2020; Alarcon-Schumacher and Erdmann, 2022; Laso-Pérez et al., 2023). However, from marine environments, only viruses of the marine planktonic archaea *Nitrosopumilus* belong to Marine Group I (MGI) archaea [currently known as phylum Thaumarchaeota or class Nitrososphaera (Parks et al., 2021)] have been isolated and studied in laboratory (Kim et al., 2019). The lack of pure cultures of marine archaea has significantly limited our understanding of the diversity, distribution, and ecological functions of archaeal viruses.

Marine Group II (MGII) archaea [currently assigned as *Ca.* Poseidoniales (Rinke et al., 2019)] are among the dominant marine archaea groups widely distributed in global oceans (Zhang et al., 2015). They are thought to be involved in heterogeneous processes in response to phytoplankton bloom and seasonal variation in organic matter composition in seawater (DeLong et al., 1994; Murray et al., 1999; Massana et al., 2000; Liu et al., 2007). They can metabolize high molecular weight organic matter such as proteins, lipids, and carbohydrates (Iverson et al., 2012; Baker et al., 2013; Deschamps et al., 2014). MGII archaea display a variety of ecological patterns and metabolic capability, suggesting their flexibility in global biogeochemical cycles (Rinke et al., 2019; Tully, 2019).

To date, no isolated or enriched cultures of MGII archaea have been obtained. The genome sequences of candidate viruses of MGII archaea were only recently identified from metagenome sequences of global open oceans and coasts (Nishimura et al., 2017; Philosof et al., 2017; Vik et al., 2017; Zhou et al., 2022). Named as magroviruses by Philosof et al. (2017), these viruses of MGII archaea belong to *Caudovirales* and possess double-stranded DNA genomes of about 90 kb on average. Phylogenetic analysis reveals that they have close evolutionary connection to viruses of haloarchaea (haloviruses). Like haloviruses, magroviruses may also have icosahedral capsids and helical tails (Philosof et al., 2017; Krupovic et al., 2018). Abundance analysis revealed that they are widespread in surface ocean water and are possibly the third most abundant marine planktonic viruses after cyanophages and pelagiphages implying their potential roles in marine ecology by interacting with their hosts (Philosof et al., 2017; Zhou et al., 2022).

Estuaries are the convergent zone of freshwater and seawater. They are characterized by a significant variety of nutrients, temperature, salinity, and other environmental factors, and inhabited by vast diversity of microorganisms (Cloern, 1987; Ait Alla et al., 2006; Cai et al., 2016; Xie et al., 2018; Sun et al., 2021; Xu et al., 2022). A recent study reported MGII archaea at the Pearl River estuary with abundance being an order of magnitude higher than previously reported in marine environments, providing a new perspective on the salinity tolerance of MGII archaea (Xie et al., 2018). Fan et al. (2022) further investigated the genomic diversity of MGII archaea in global brackish environments and showed that brackish-specific MGII archaea possessed distinct evolutionary and ecological features compared to their commonly known marine relatives. However, the genomic features and ecological functions of magroviruses are unknown in estuaries and other brackish environments.

This study aims to identify the diversity, spatial distribution, and genome features of magroviruses in metagenomes of brackish environments. 234 non-redundant novel magroviral genome sequences were derived and phylogenetic tree of marker genes revealed six taxonomic group of global magroviruses. Distinct distribution of marine and brackish magroviruses in habitats with different salinity was observed and genome annotation implies their diverse ecological potentials by interacting with the metabolism of their archaeal hosts.

## Materials and methods

### The curation of a marine reference dataset of magroviruses

We collected 84 genome sequences of magroviruses from previous studies with a length range from 22.55 to 120.96 kb (Nishimura et al., 2017; Philosof et al., 2017) to construct a non-redundant reference dataset of marine magroviruses. Five (Ahlgren et al., 2019) and 46 (Liu et al., 2021) head tail viruses infecting MGI archaea and haloarchaea, respectively, were added as the outgroup. The protein sequence of major capsid protein (MCP) and DNA polymerase B (DNApolB) genes of these reference genomes were extracted according to the annotation information reported by Nishimura et al. (2017) and Philosof et al. (2017), and used as queries to obtain homologous genes from the candidate viral genomes by using Prodigal (Hyatt et al., 2010) and PSI-BLAST (evalue = 1e-05, max_target_seqs = 1e-07) (Altschul et al., 1990).

Moreover, an extra step was used to expand magroviral sequences in this dataset using the following procedure. Firstly, 43 candidate genome sequences of archaeal viruses may infecting MGI and MGII archaea were obtained from a metagenome study specifically targeting archaeal viruses in marine surface water (Vik et al., 2017). Then, the phylogenetic trees of MCP and DNApolB were reconstructed with the corresponded reference sequences of magroviruses, MGI viruses and haloviruses, respectively, to identify 20 magroviruses by manual check. Finally, all magroviral sequences were pooled and dereplicated by using cd-hit-est (v4.6.8, -c 0.99 -aS 0.99) (Fu et al., 2012) resulting a total of 104 reference genomes of marine magroviruses with a length range from 10.05 to 120.96 kb (Supplementary Table S1).

### Identification of magroviruses from brackish environments

The clean reads and metagenomic assemblies were collected from previous studies including the metagenome of the Pearl River estuary (PRE) (Fan et al., 2022; Xu et al., 2022), the Yaquina Bay estuary (YBE) (Kieft et al., 2018), the Caspian Sea (CPS) (Mehrshad et al., 2016), and global oceanic samples (Brum et al., 2015; Pesant et al., 2015; Supplementary Table S2). Contigs longer than 5 kb were piped through VirSorter (categories 1–6) (Roux et al., 2015), VirSorter2 (score > 0.7) (Guo et al., 2021), VirFinder (score > 0.7 and *p* < 0.05) (Ren et al., 2017) and DeepVirFinder (score > 0.7 and *p* < 0.05) (Ren et al., 2020) to identify putative viral genome sequences. Contigs that assigned to categories with the most confident (categories 1 and 4) or likely (categories 2 and 5) predictions by VirSorter, or with max score > 0.9 predicted by VirSorter2, or with score > 0.9 and *p* < 0.05 predicted by VirFinder or DeepVirFinder were directly classified as viral. For the remaining contigs, only those identified by two or more methods or having 40% of genes classified as viruses by CAT were kept (von Meijenfeldt et al., 2019).

A total of 187,125 viral genome sequences were identified. For each viral genome, genes were called by using Prodigal. Protein sequences of these genes were then searched by using PSI-BLAST (evalue = 1e-05 and max_target_seqs = 1e-07) against the reference sequence of MCP and DNApolB gene as queries resulting a total of 304 candidate magroviral genomes. Finally, cd-hit-est (v4.6.8, -c 0.99 -aS 0.99) were applied to remove the redundant sequences. There was no redundancy observed between the brackish magroviral genome dataset and the marine reference dataset based on a further cd-hit-est analysis.

### Phylogenetic analysis

The protein sequences of MCP and DNApolB, respectively, were used to conduct phylogenetic analysis of magroviruses. Specifically, the sequences of reference and brackish magroviruses were combined and aligned by using MAFFT v6 (Yamada et al., 2016), which was followed by the removal of poorly aligned positions by using trimAL (v1.2rev59; -automated1) (Capella-Gutiérrez et al., 2009). The phylogenetic trees were constructed by using FastTree (v2.1.10) with default parameters (Price et al., 2010) and visualized in the Interactive Tree of Life (iTOL, v.5.1.1) (Letunic and Bork, 2021).

### Viral genome clustering

A gene-sharing network of magroviral genomes and other prokaryotic virus genomes were conducted by using vConTACT v2.0 (Bin Jang et al., 2019). Specifically, magroviral genomes longer than 10 kb from the reference dataset and the brackish dataset were pooled and grouped into viral operational taxonomic units (vOTUs) by using the pipeline of CheckV (v1.0.1; 95% pairwise average nucleotide identity and 85% alignment fraction) (Nayfach et al., 2021). The quality of vOTUs were assessed by using CheckV. The protein sequences of the vOTUs were clustered with viral genomes from the Viral RefSeq release 201 database by using vConTACT. The virus network was visualized by using Cytoscape (v3.8.0) (Shannon et al., 2003).

### Viral genome annotation

Proteins of magroviral vOTUs were annotated based on the KEGG database by using kofamscan (Aramaki et al., 2020), the COG (Tatusov et al., 2000), arCOG (Makarova et al., 2015) and Tigrfam (Haft et al., 2003) databases by using BLASTp (E-value 1e-05, bit score > 50, similarity >30%, and coverage >50%), and the Pfam database (Finn et al., 2016) by using hmmsearch (E-value 1e-05) (Finn et al., 2011), respectively. The auxiliary metabolic genes (AMGs) were identified by using VIBRANT (v. 1.2.1) (Kieft et al., 2020) and their functional annotations were manually checked.

### Abundance analysis

To investigate the distribution of magroviruses in global marine surface water, the clean reads of metagenomes from the PRE, the YBE, the CPS, and the open ocean (Supplementary Table S2) were mapped to the vOTUs of magroviruses by using similar methods as described by Fan et al. (2022). In brief, the clean reads were first mapped to the vOTUs by using Bowtie2 (v. 2.3.5) (Langmead and Salzberg, 2012) and followed by sorting and format convert to BAM files by using SAMtools (v. 1.9) (Li et al., 2009). Then, the BAM files were filtered by using BamM (v. 1.7.3)^1^ with thresholds of 99% identity and 75% coverage. Finally, the reads per kbp of each genome per mbp of each metagenomic sample (RPKM) value was calculated by using bbmap.^2^

## Results

### The diversity and phylogenetic analysis of brackish and marine magroviruses

Our study recovered a remarkable diversity of magroviruses from brackish environments. A total of 234 non-redundant magroviral genome sequences were obtained from the metagenomes of two estuaries and one enclosed sea and their length ranges from 5.1 kb to 103 kb (Supplementary Table S1). 184, 44, and 6 magroviral genomes were obtained from the PRE, the YBE and the CPS, respectively. There was no redundancy (ANI = 99%) in magroviral genomes between the brackish and marine environment datasets. Most PRE magroviruses (*n* = 146; 79%) were obtained from the large particulate size (microbial cellular) fraction of planktonic samples (i.e., particulate size >0.22 μm), implying these magroviruses were either in the intracellular state of their lifecycle or attached to microbial cells or aggregates.

**Figure 1:**
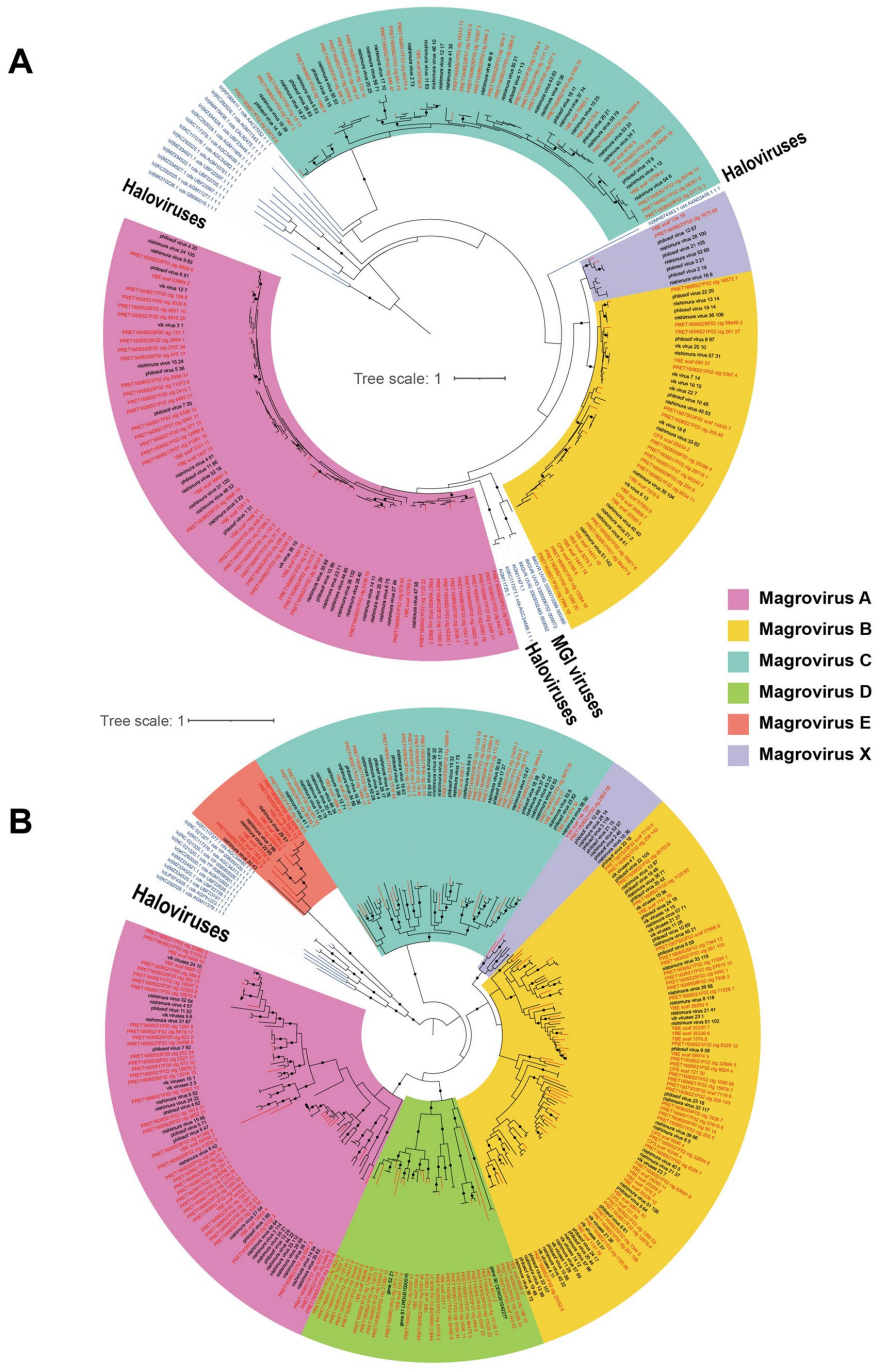
Unrooted maximum-likelihood trees of MCP **(A)** and DNApolB **(B)**, respectively. The names of brackish magroviruses are in red, the marine references are in black, and the other tailed viruses are in blue. The solid dots on internal branches show branch supports >0.95 (based on 1,000 iterations of bootstrapping). The colored blocks show subgroups of magroviruses.

The phylogenetic trees of magroviruses were reconstructed based on the protein sequences of MCP and DNApolB, respectively, to assess their evolutionary diversity. Six groups were identified in these two trees including group A, B, C, D, E, and X (Figure 1). In the MCP tree, magroviruses split into two clades: one was group C, which joins in the clade of haloviruses with a long branch; the other consisting of group A, B and X formed a mixed clade with halovirus and MGI viruses, suggesting its close evolutionary relationship with archaeal tailed viruses (Figure 1A). The topological relationship of groups A, B, C, and X were consistent between these two trees. However, group D and E were only found in the DNApolB tree, suggesting may only a subgroup of magroviruses encoding the MCP gene. Haloviruses formed a sister-group of all magroviruses in the DNApolB tree, which showed a similar pattern as previously reported (Philosof et al., 2017; Figure 1B). The brackish magroviruses were found in all the six groups and enriched in groups A and D, showing no distinction between brackish- or marine- specific branches.

Magroviral genomes from the marine and brackish datasets were pooled and clustered to 228 vOTUs. Notably, only 2 out of 228 vOTUs contained magroviral genomes from both brackish and marine environments.

### The genus-level taxonomic assignment of magroviruses

**Figure 2:**
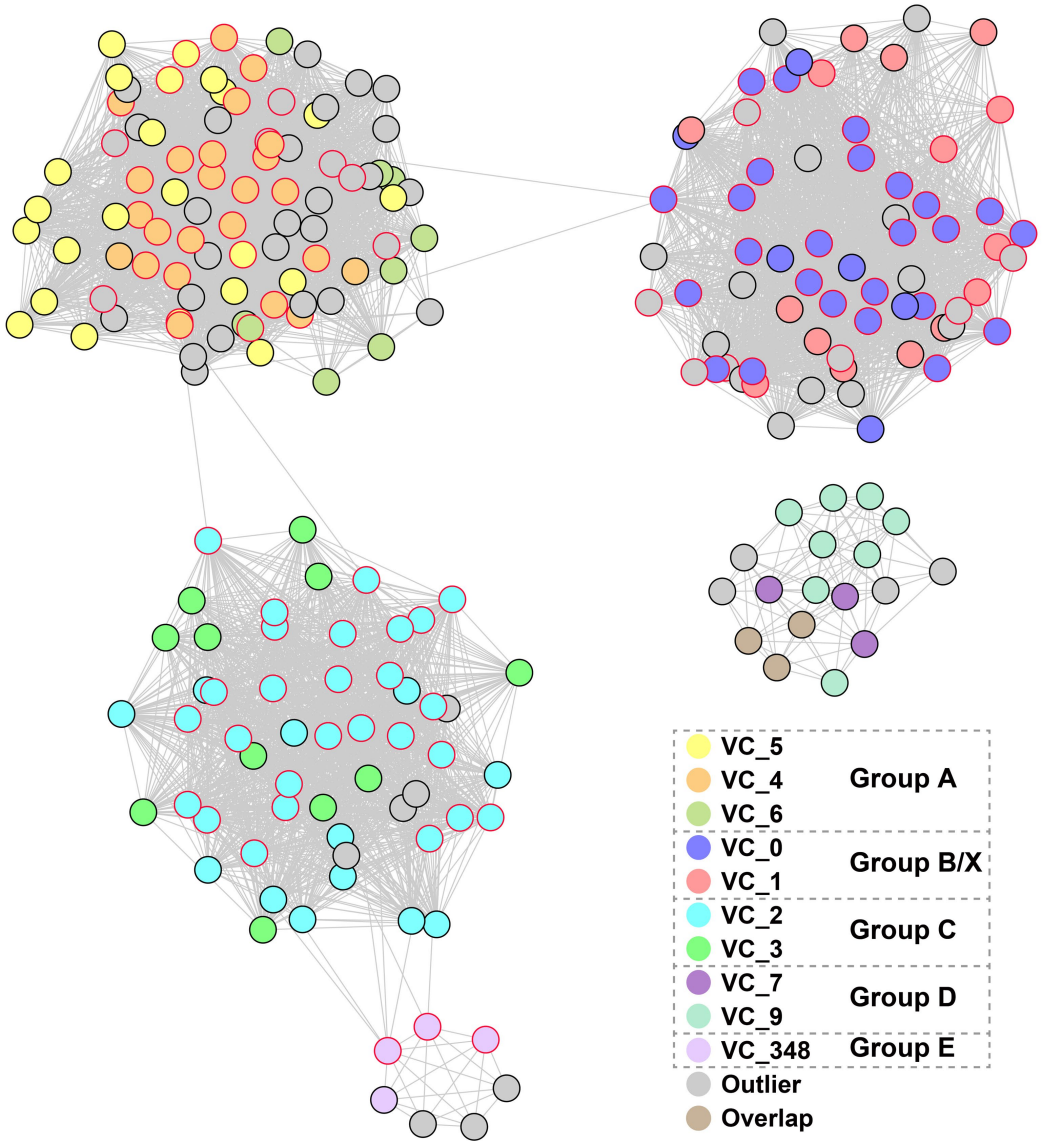
The network-based analysis of PCs shared among magroviral vOTUs. The nodes represent vOTUs, and the edges represent the strength of connectivity between each genome based on shared PCs.

Because of the lack of universal marker genes in viruses for reliable evolutionary analysis, trees of MCP and DNApolB can be biased by the genes’ own evolutionary histories. To further assess the diversity of magrovirus and the evolutionary relationship between magroviruses and other archaeal head-tailed viruses, we conducted an approximately genus-level operational taxonomic assignment of magroviral vOTUs using vConTACT v2.0. According to the protein clusters (PCs) sharing networks, all magroviral vOTUs were embedded together resulting in five large clusters, which were disconnected from all non-magroviral clusters (Supplementary Figure S1). These five large magroviral clusters and can be further divided into ten viral clusters (VCs) (Figure 2). By comparing the trees of MCP, DNApolB and the results of vConTACT analysis, we found that phylogenetic group A can be subdivided into three VCs including VC_4, VC_5 and VC_6. The vOTUs of group B and X were mixed and consist of VC_0 and VC_1. Both group C and D are formed two VCs, the former consist of VC_2 and VC_3, whereas the latter including VC_7 and VC_9. Group E was only composed of VC_348. Noteworthy, some vOTUs were defined as outliers or assigned to multiple VCs possibly as the result of their fragmented genomes.

### The distribution of magroviruses in marine and brackish environments

**Figure 3:**
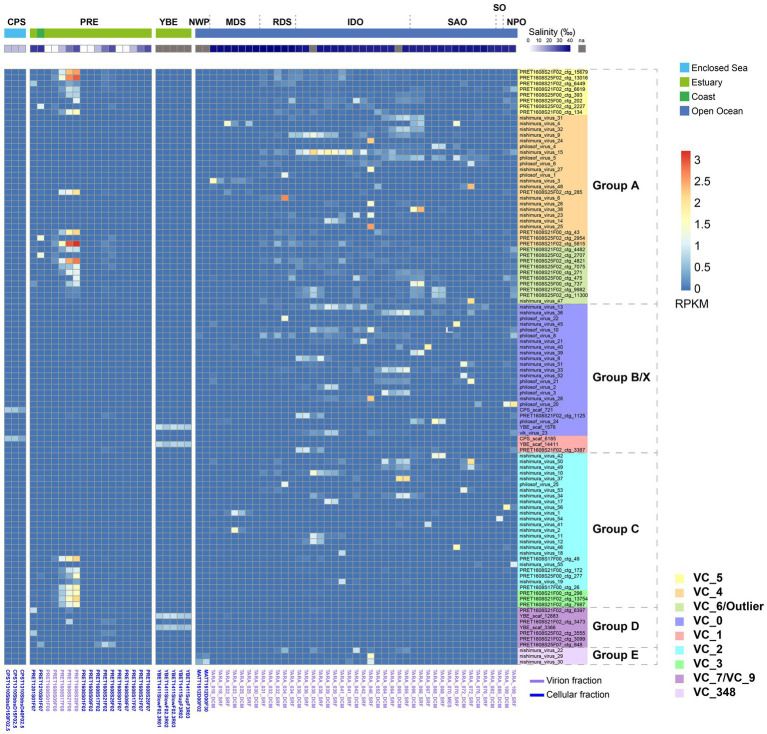
Global abundance and distribution of magroviruses. Heatmap shows the magroviral vOTUs with max RPKM value above 0.9. Abbreviations of sampling areas: CPS, Caspian Sea; PRE, Pearl River estuary; YBE, Yaquina Bay estuary; NWP, Northwest Pacific Ocean; MDS, Mediterranean Sea; RDS, Read Sea; IDO, Indian Ocean; SAO, South Atlantic Ocean; SO, Southern Ocean; NPO, North Pacific Ocean. Colors of VCs are consistent to Figure 2.

We calculated the abundance of magroviral vOTUs in brackish (CPS, PRE, and YBE) and marine metagenomes (Figure 3; Supplementary Table S2). Magroviruses widely distributed in high abundance both in brackish and marine environments, but the patterns of distribution were different between phylogenetic groups. Specifically, viruses in group A and C were found in high abundance in both brackish and marine samples. Most viruses in group B and E were enriched in marine environments, while those of VC_3 were more abundant in brackish environments.

In the 103 most abundant (maximum RPKM in samples >0.9) vOTUs, 52 were present (RPKM >0.01) only in marine samples and three in brackish samples (Figure 3). These magroviruses were considered as salinity specific. The remaining 48 are found in both marine and brackish. They were therefore assigned as salinity broad-spectrum lineages. Interestingly, almost all magroviral vOTUs from the brackish dataset (91.4%) were salinity broad-spectrum. In contrast, most vOTUs of the marine dataset (76.5%) were only detected in marine environments.

Two vOTUs belonging to group B were abundant in the CPS (Figure 3). In the YBE, four vOTUs belonging to group B and D were the most abundant magroviruses. In contrast, a highly diverse community of magroviruses from all groups except D were found abundant in the PRE in consistent to reported high diversity of MGII archaea in this estuary (Fan et al., 2022). They were generally much more abundant in the virion fractions (i.e., particulate size <0.22 μm) than in the microbial cellular fractions, possibly suggesting a lytic lifestyle of magroviruses in this environment. The only exception was group D, which was more enriched in the microbial cellular fractions. In the PRE, the abundance of almost all vOTUs increases along with the increasing salinity (Figure 3) sharing a similar distribution pattern of marine subgroups of MGII archaea as we previously reported (Fan et al., 2022).

Notably, while most VCs contained both brackish and marine magroviruses, VC_3, VC_5, VC_7, VC_9, and VC_4 had over twice the number of brackish viruses than marine ones, while VC_6 had over twice the number of marine ones than the brackish ones. VC_3 contained exclusively brackish relatives. This observation suggests biased distribution of these genera in brackish and marine environments.

### Genome organization of magroviruses

**Figure 4:**
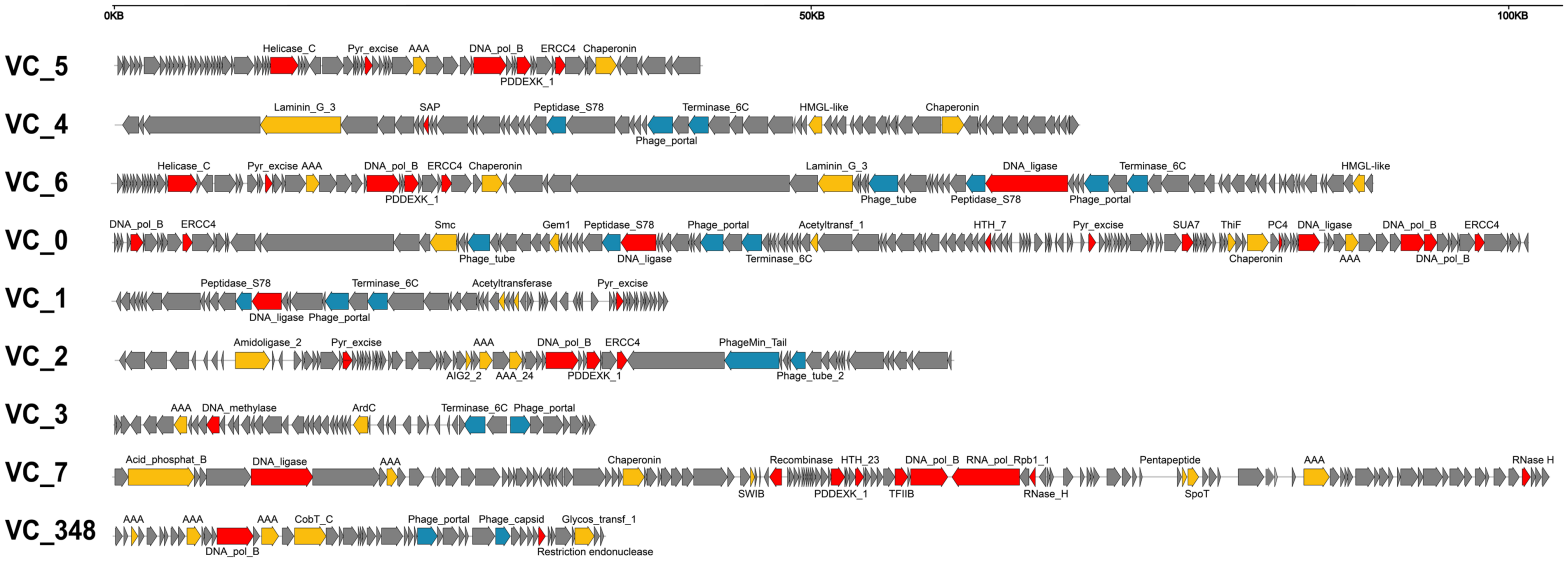
Summary of representative genome organization in different VCs of magroviruses. Different colors indicate genes with different functional categories: DNA metabolism genes are in red, structural genes are in blue, other functional genes are in yellow, and unclassified genes are in grey.

We found no specific pattern of genome organization in brackish magroviruses in comparison to marine ones. Magroviral genome generally consisted of replicative module, structural module, and other metabolic blocks (Figure 4). Almost all the vOTUs encoded DNApolB, DNA ligase, ERCC4 type nuclease, and DNA glycosylase, representing an expansive suite of almost complete replication protein blocks. Except for group D and VC_5 of group A, most magroviral genomes encoded structural proteins including phage portal protein, terminase, major capsid protein, caudovirus prohead serine protease, and minor tail proteins (Figure 4). The absence of structural genes in group D and VC_5 was also reported by Vik et al. (2017) and Philosof et al. (2017), respectively. While this observation could be explained by possible incomplete genomes of viruses in these groups, or by poor gene annotation, another possibility is that group D and VC_5 may be a class of plasmid-like mobile gene elements. However, further evidence is required to verify these assumptions.

### Metabolic potentials of magroviruses

**Figure 5:**
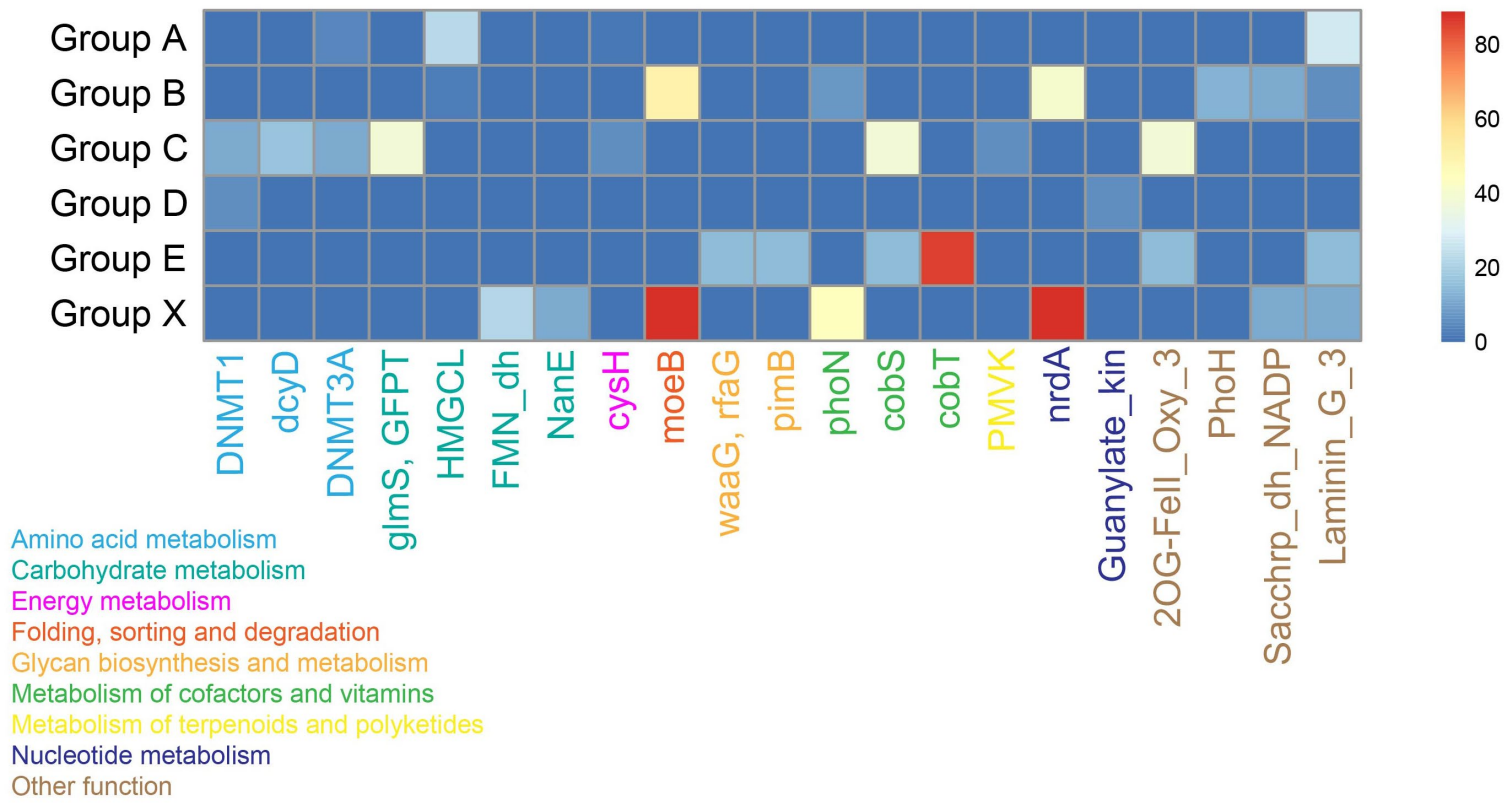
Heatmap shows the presence of AMGs in magroviral groups. Color density shows the percentage of magroviral genome encoding the AMG in each group.

Magroviruses encoded diverse AMGs involving in KEGG categories of ‘Amino acid metabolism’, ‘Carbohydrate metabolism’, ‘Energy metabolism’, ‘Folding, sorting and degradation’, ‘Glycan biosynthesis and metabolism’, ‘Metabolism of cofactors and vitamins’, ‘Metabolism of terpenoids and polyketides’, and ‘Nucleotide metabolism’ (Figure 5; Supplementary Table S3). The carbohydrate metabolism was the predominance AMGs encoded by magroviruses, including glutamine-fructose-6-phosphate transaminase (GlmS) involving in UDP-N-acetyl-D-glucosamine biosynthesis, hydroxymethylglutaryl-CoA lyase (HMGCL) involving in leucine degradation, (S)-2-hydroxy-acid oxidase (FMN_dh) and N-acylglucosamine-6-phosphate 2-epimerase (NanE) involving in amino sugar and nucleotide sugar metabolism, followed by metabolism of cofactors and vitamins including acid phosphatase (class A) (PhoN), cobaltochelatase CobS and CobT involving in riboflavin biosynthesis, and cobalamin. Some magroviral genomes encoded genes involving in sulfur, phosphorus, and iron elemental cycle including phosphoadenosine phosphosulfate reductase (CysH), PhoH-like protein (PhoH), and 2OG-Fe (II) oxygenase superfamily (2OG-FeII_Oxy_3).

Notably, these AMGs are sporadically distributed among vOTUs. Most of AMGs were present in no more than two groups and only concanavalin A-like lectin/glucanases superfamily (Laminin_G_3) was found in four groups. Few AMGs were observed in group D.

## Discussion

In this study, we expanded the genome dataset of global magroviruses by identifying a vast diversity of this viral lineage from brackish environments including estuaries and an enclosed sea. This expanded dataset provides us an opportunity to validate the evolutionary relationship between magroviruses and other archaeal viruses.

Previous studies showed close relationship between magroviruses and tailed haloviruses (Philosof et al., 2017; Krupovic et al., 2018). Here, we further identified the evolutionary connection between magroviruses and MGI archaea viruses. In the MCP tree, magroviruses formed a monophyletic clade with haloviruses and this clade was in sister-relationship with MGI archaea viruses (Figure 1A). Thus, these closely related *Caudovirales* viruses as inferred by Krupovic et al. (2018) may have similar head-tailed structure and share a common ancestor. We notice that some magroviral genomes were incomplete or in low quality according to the CheckV assessment possibly as the result of low data coverage and assembly difficulty (Supplementary Table S1). Therefore, it is hard in this study to provide a reliable taxonomic assignment and ranking of magroviruses as what has been done for haloviruses (Liu et al., 2021). Future approaches with long-read sequencing data may help recover complete genomes of magroviruses.

The distribution of magroviruses in the PRE salinity gradient generally follows a salinity-preferred pattern: more abundant magroviruses were found in higher salinity while magroviral abundance was substantially depleted in salinity below 10‰ and none magroviral sequences were detected below salinity 1.17‰ (Figure 3). This distribution pattern is consistent to the distribution of marine subgroups of MGII archaea as shown in our previous study (Fan et al., 2022), suggesting that marine MGII archaea with preference of high salinity (> 30‰) may be the potential hosts of magroviruses in the PRE. However, more evidence such as CRISPR or provirus information is needed to further test this hypothesis.

Phylogenetic analysis in this study generally failed to identify subclades of magroviruses specifically distributing in brackish- or marine- waters, suggesting possible frequent marine-brackish transitions in evolution of this viral clade (Figure 3). However, vOTUs do show variation in different habitats (i.e., oceans, estuaries or enclosed seas). While some vOTUs were abundant only in metagenomes of open oceans, others could be detected both in ocean and PRE samples. Interestingly, almost all magroviral vOTUs from the brackish dataset (91.4%) have a broad-spectrum of habitat types (i.e., eurychoric viruses), suggesting magroviruses or their archaeal hosts inhabiting brackish environments are usually able to tolerate a broad-spectrum of salinity and thus may also live in marine environments.

The discovery of diverse AMGs in magroviruses suggests previously underestimated metabolic potentials of MGII archaea. Specifically, magroviruses encoding AMGs involving in substrate degradation may facilitate their hosts in degrading high molecular weight organic matter for nutrients and energy (Zhang et al., 2021). The *cysH* gene encoding the phosphoadenosine 5′-phosphosulfate reductase functions in biosynthesis of sulfite by assimilatory sulfate reduction (Bick et al., 2000). Magroviruses encoding this gene may serve as an important supplement of sulfur assimilation of MGII archaea (Santoro et al., 2019). Moreover, MGII archaea infected by viruses containing the phosphate uptake regulation gene *phoH* may be aided in surviving in low phosphate seawater (Goldsmith et al., 2011). Intriguingly, MGII archaea were previously considered as lacking the capability to synthesize cobalamin and biotin and they may need to acquire these vitamins from other microbes, such as thaumarchaea and cyanobacteria (Iverson et al., 2012; Santoro et al., 2019). However, the discovery of cobalamin and biotin biosynthesis genes in magroviruses suggests MGII archaea infected by these viruses may obtain such vitamins by themselves.

The wide occurrence and metabolic potential of magroviruses discovered in this study suggest a potential global impact of magroviruses on the ecological functions of MGII archaea. During the manuscript preparation of this study, Zhou et al. (2022) published a paper on the metagenome sequences of archaeal viruses, which included magroviruses from a coastal but not brackish zone. Our finding of the brackish magroviruses and that of Zhou et al. (2022) indicate a substantial diversity of magroviruses on coastal environments.

## Data availability statement

The original contributions presented in this study are included in the article/Supplementary material, further inquiries can be directed to the corresponding author.

## Author contributions

LF and CZ conceived this study. BX, LF, WW, and YZ collected the data. BX analyzed the metagenome data, produced the genomes, and conducted all other analyses. BX, LF, and WW interpreted the results and drafted the manuscript. All authors contributed to the final version of the manuscript.

## Funding

This study was supported by the National Key Research and Development Program of China (2018YFA0605802), the National Natural Science Foundation of China (91951120, 91851210, and 42141003), the Open Project of Key Laboratory of Environmental Biotechnology, CAS (KF2021006), the Department of Science and Technology of Guangdong Province (2021B1515120080), the Shenzhen Science and Technology Innovation Commission (ZDSYS201802081843490), the Southern Marine Science and Engineering Guangdong Laboratory (Guangzhou) (2018B030311016) and the Shanghai Sheshan National Geophysical Observatory (2020Z01). Computation in this study was supported by the Centre for Computational Science and Engineering at the Southern University of Science and Technology.

## Conflict of interest

The authors declare that the research was conducted in the absence of any commercial or financial relationships that could be construed as a potential conflict of interest.

## Publisher’s note

All claims expressed in this article are solely those of the authors and do not necessarily represent those of their affiliated organizations, or those of the publisher, the editors and the reviewers. Any product that may be evaluated in this article, or claim that may be made by its manufacturer, is not guaranteed or endorsed by the publisher.

## Supplementary material

The Supplementary material for this article can be found online at: https://www.frontiersin.org/articles/10.3389/fmicb.2023.1151034/full#supplementary-material

## Footnotes

^1^ https://github.com/minillinim/BamM

^2^ http://jgi.doe.gov/data-and-tools/bb-tools/

## References

[ref1] Ahlgren N. A. Fuchsman C. A. Rocap G. Fuhrman J. A. (2019). Discovery of several novel, widespread, and ecologically distinct marine Thaumarchaeota viruses that encode amoC nitrification genes. ISME J. 13, 618–631. doi: 10.1038/s41396-018-0289-4, PMID: 30315316 PMC6462027

[ref2] Ait Alla A. Mouneyrac C. Durou C. Moukrim A. Pellerin J. (2006). Tolerance and biomarkers as useful tools for assessing environmental quality in the Oued Souss estuary (bay of Agadir, Morocco). Comp. Biochem. Physiol. C Toxicol. Pharmacol. 143, 23–29. doi: 10.1016/j.cbpc.2005.11.015, PMID: 16413830

[ref3] Alarcon-Schumacher T. Erdmann S. (2022). A trove of Asgard archaeal viruses. Nat. Microbiol. 7, 931–932. doi: 10.1038/s41564-022-01148-2, PMID: 35760838

[ref4] Altschul S. F. Gish W. Miller W. Myers E. W. Lipman D. J. (1990). Basic local alignment search tool. J. Mol. Biol. 215, 403–410. doi: 10.1016/S0022-2836(05)80360-2 2231712

[ref5] Aramaki T. Blanc-Mathieu R. Endo H. Ohkubo K. Kanehisa M. Goto S.. (2020). KofamKOALA: KEGG Ortholog assignment based on profile HMM and adaptive score threshold. Bioinformatics 36, 2251–2252. doi: 10.1093/bioinformatics/btz859, PMID: 31742321 PMC7141845

[ref6] Baker B. J. Sheik C. S. Taylor C. A. Jain S. Bhasi A. Cavalcoli J. D.. (2013). Community transcriptomic assembly reveals microbes that contribute to deep-sea carbon and nitrogen cycling. ISME J. 7, 1962–1973. doi: 10.1038/ismej.2013.85, PMID: 23702516 PMC3965313

[ref7] Baquero D. P. Contursi P. Piochi M. Bartolucci S. Liu Y. Cvirkaite-Krupovic V.. (2020). New virus isolates from Italian hydrothermal environments underscore the biogeographic pattern in archaeal virus communities. ISME J. 14, 1821–1833. doi: 10.1038/s41396-020-0653-z, PMID: 32322010 PMC7305311

[ref8] Bick J. A. Dennis J. J. Zylstra G. J. Nowack J. Leustek T. (2000). Identification of a new class of 5′-adenylylsulfate (APS) reductases from sulfate-assimilating bacteria. J. Bacteriol. 182, 135–142. doi: 10.1128/JB.182.1.135-142.2000, PMID: 10613872 PMC94249

[ref9] Bin Jang H. Bolduc B. Zablocki O. Kuhn J. H. Roux S. Adriaenssens E. M.. (2019). Taxonomic assignment of uncultivated prokaryotic virus genomes is enabled by gene-sharing networks. Nat. Biotechnol. 37, 632–639. doi: 10.1038/s41587-019-0100-8, PMID: 31061483

[ref10] Brum J. R. Ignacio-Espinoza J. C. Roux S. Doulcier G. Acinas S. G. Alberti A.. (2015). Ocean plankton. Patterns and ecological drivers of ocean viral communities. Science 348:1261498. doi: 10.1126/science.1261498, PMID: 25999515

[ref11] Cai L. Zhang R. He Y. Feng X. Jiao N. (2016). Metagenomic analysis of Virioplankton of the subtropical Jiulong River estuary, China. Viruses 8:35. doi: 10.3390/v8020035, PMID: 26848678 PMC4776190

[ref12] Capella-Gutiérrez S. Silla-Martínez J. M. Gabaldón T. (2009). trimAl: a tool for automated alignment trimming in large-scale phylogenetic analyses. Bioinformatics 25, 1972–1973. doi: 10.1093/bioinformatics/btp348, PMID: 19505945 PMC2712344

[ref13] Cloern J. E. (1987). Turbidity as a control on phytoplankton biomass and productivity in estuaries. Cont. Shelf Res. 7, 1367–1381. doi: 10.1016/0278-4343(87)90042-2

[ref14] Delong E. F. Wu K. Y. Prezelin B. B. Jovine R. V. (1994). High abundance of archaea in Antarctic marine picoplankton. Nature 371, 695–697. doi: 10.1038/371695a0, PMID: 7935813

[ref15] Deschamps P. Zivanovic Y. Moreira D. Rodriguez-Valera F. Lopez-Garcia P. (2014). Pangenome evidence for extensive interdomain horizontal transfer affecting lineage core and shell genes in uncultured planktonic thaumarchaeota and euryarchaeota. Genome Biol. Evol. 6, 1549–1563. doi: 10.1093/gbe/evu127, PMID: 24923324 PMC4122925

[ref16] Fan L. Xu B. Chen S. Liu Y. Li F. Xie W.. (2022). *CorA* gene rearrangement triggered the salinity-driven speciation of Poseidoniales. bioRxiv. doi: 10.1101/2022.09.25.509439

[ref17] Finn R. D. Clements J. Eddy S. R. (2011). HMMER web server: interactive sequence similarity searching. Nucleic Acids Res. 39, W29–W37. doi: 10.1093/nar/gkr367, PMID: 21593126 PMC3125773

[ref18] Finn R. D. Coggill P. Eberhardt R. Y. Eddy S. R. Mistry J. Mitchell A. L.. (2016). The Pfam protein families database: towards a more sustainable future. Nucleic Acids Res. 44, D279–D285. doi: 10.1093/nar/gkv1344, PMID: 26673716 PMC4702930

[ref001] Fu L. Niu B. Zhu Z. Wu S. Li W. (2012). CD-HIT: accelerated for clustering the next-generation sequencing data. Bioinformatics 28, 3150–3152. doi: 10.1093/bioinformatics/bts565 23060610 PMC3516142

[ref19] Goldsmith D. B. Crosti G. Dwivedi B. McDaniel L. D. Varsani A. Suttle C. A.. (2011). Development of phoH as a novel signature gene for assessing marine phage diversity. Appl. Environ. Microbiol. 77, 7730–7739. doi: 10.1128/AEM.05531-11, PMID: 21926220 PMC3209181

[ref20] Guo J. Bolduc B. Zayed A. A. Varsani A. Dominguez-Huerta G. Delmont T. O.. (2021). VirSorter2: a multi-classifier, expert-guided approach to detect diverse DNA and RNA viruses. Microbiome 9:37. doi: 10.1186/s40168-020-00990-y, PMID: 33522966 PMC7852108

[ref21] Haft D. H. Selengut J. D. White O. (2003). The TIGRFAMs database of protein families. Nucleic Acids Res. 31, 371–373. doi: 10.1093/nar/gkg128, PMID: 12520025 PMC165575

[ref22] Hyatt D. Chen G. L. Locascio P. F. Land M. L. Larimer F. W. Hauser L. J. (2010). Prodigal: prokaryotic gene recognition and translation initiation site identification. BMC Bioinformat. 11:119. doi: 10.1186/1471-2105-11-119, PMID: 20211023 PMC2848648

[ref23] Iverson V. Morris R. M. Frazar C. D. Berthiaume C. T. Morales R. L. Armbrust E. V. (2012). Untangling genomes from metagenomes: revealing an uncultured class of marine Euryarchaeota. Science 335, 587–590. doi: 10.1126/science.1212665, PMID: 22301318

[ref24] Kieft B. Li Z. Bryson S. Crump B. C. Hettich R. Pan C.. (2018). Microbial community structure-function relationships in Yaquina Bay estuary reveal spatially distinct carbon and nitrogen cycling capacities. Front. Microbiol. 9:1282. doi: 10.3389/fmicb.2018.01282, PMID: 29963029 PMC6010575

[ref25] Kieft K. Zhou Z. Anantharaman K. (2020). VIBRANT: automated recovery, annotation and curation of microbial viruses, and evaluation of viral community function from genomic sequences. Microbiome 8:90. doi: 10.1186/s40168-020-00867-0, PMID: 32522236 PMC7288430

[ref26] Kim J. G. Kim S. J. Cvirkaite-Krupovic V. Yu W. J. Gwak J. H. López-Pérez M.. (2019). Spindle-shaped viruses infect marine ammonia-oxidizing thaumarchaea. Proc. Natl. Acad. Sci. U. S. A. 116, 15645–15650. doi: 10.1073/pnas.1905682116, PMID: 31311861 PMC6681747

[ref27] Krupovic M. Cvirkaite-Krupovic V. Iranzo J. Prangishvili D. Koonin E. V. (2018). Viruses of archaea: structural, functional, environmental and evolutionary genomics. Virus Res. 244, 181–193. doi: 10.1016/j.virusres.2017.11.025, PMID: 29175107 PMC5801132

[ref28] Langmead B. Salzberg S. L. (2012). Fast gapped-read alignment with bowtie 2. Nat. Methods 9, 357–359. doi: 10.1038/nmeth.1923, PMID: 22388286 PMC3322381

[ref29] Laso-Pérez R. Wu F. Crémière A. Speth D. R. Magyar J. S. Zhao K.. (2023). Evolutionary diversification of methanotrophic ANME-1 archaea and their expansive virome. Nat. Microbiol. 8, 231–245. doi: 10.1038/s41564-022-01297-4, PMID: 36658397 PMC9894754

[ref30] Letunic I. Bork P. (2021). Interactive tree of life (iTOL) v5: an online tool for phylogenetic tree display and annotation. Nucleic Acids Res. 49, W293–W296. doi: 10.1093/nar/gkab301, PMID: 33885785 PMC8265157

[ref31] Li H. Handsaker B. Wysoker A. Fennell T. Ruan J. Homer N.. (2009). The sequence alignment/map format and SAMtools. Bioinformatics 25, 2078–2079. doi: 10.1093/bioinformatics/btp352, PMID: 19505943 PMC2723002

[ref32] Liu H. B. Chang J. Tseng C. M. Wen L. S. Liu K. K. (2007). Seasonal variability of picoplankton in the northern South China Sea at the SEATS station. Deep Sea Res II Topical Stud. Oceanograph. 54, 1602–1616. doi: 10.1016/j.dsr2.2007.05.004

[ref33] Liu Y. Demina T. A. Roux S. Aiewsakun P. Kazlauskas D. Simmonds P.. (2021). Diversity, taxonomy, and evolution of archaeal viruses of the class Caudoviricetes. PLoS Biol. 19:e3001442. doi: 10.1371/journal.pbio.3001442, PMID: 34752450 PMC8651126

[ref34] Makarova K. S. Wolf Y. I. Koonin E. V. (2015). Archaeal clusters of orthologous genes (arCOGs): an update and application for analysis of shared features between Thermococcales, Methanococcales, and Methanobacteriales. Life (Basel) 5, 818–840. doi: 10.3390/life5010818, PMID: 25764277 PMC4390880

[ref35] Massana R. Delong E. F. Pedros-Alio C. (2000). A few cosmopolitan phylotypes dominate planktonic archaeal assemblages in widely different oceanic provinces. Appl. Environ. Microbiol. 66, 1777–1787. doi: 10.1128/AEM.66.5.1777-1787.2000, PMID: 10788339 PMC101412

[ref36] Mehrshad M. Amoozegar M. A. Ghai R. Shahzadeh Fazeli S. A. Rodriguez-Valera F. (2016). Genome reconstruction from metagenomic data sets reveals novel microbes in the brackish waters of the Caspian Sea. Appl. Environ. Microbiol. 82, 1599–1612. doi: 10.1128/AEM.03381-15, PMID: 26729711 PMC4771326

[ref37] Murray A. E. Blakis A. Massana R. Strawzewski S. Passow U. Alldredge A.. (1999). A time series assessment of planktonic archaeal variability in the Santa Barbara Channel. Aquat. Microb. Ecol. 20, 129–145. doi: 10.3354/ame020129

[ref38] Nayfach S. Camargo A. P. Schulz F. Eloe-Fadrosh E. Roux S. Kyrpides N. C. (2021). CheckV assesses the quality and completeness of metagenome-assembled viral genomes. Nat. Biotechnol. 39, 578–585. doi: 10.1038/s41587-020-00774-7, PMID: 33349699 PMC8116208

[ref39] Nishimura Y. Watai H. Honda T. Mihara T. Omae K. Roux S.. (2017). Environmental viral genomes shed new light on virus-host interactions in the ocean. ASM J. 2, e00359–e00316. doi: 10.1128/mSphere.00359-16, PMID: 28261669 PMC5332604

[ref40] Parks D. H. Chuvochina M. Rinke C. Mussig A. J. Chaumeil P.-A. Hugenholtz P. (2021). GTDB: an ongoing census of bacterial and archaeal diversity through a phylogenetically consistent, rank normalized and complete genome-based taxonomy. Nucleic Acids Res. 50, D785–D794. doi: 10.1093/nar/gkab776%JNucleicAcidsResearch PMC8728215 34520557

[ref41] Pesant S. Not F. Picheral M. Kandels-Lewis S. Le Bescot N. Gorsky G.. (2015). Open science resources for the discovery and analysis of Tara oceans data. Sci Data 2:150023. doi: 10.1038/sdata.2015.23, PMID: 26029378 PMC4443879

[ref42] Philosof A. Yutin N. Flores-Uribe J. Sharon I. Koonin E. V. Beja O. (2017). Novel abundant oceanic viruses of uncultured marine group II Euryarchaeota. Curr. Biol. 27, 1362–1368. doi: 10.1016/j.cub.2017.03.052, PMID: 28457865 PMC5434244

[ref43] Price M. N. Dehal P. S. Arkin A. P. (2010). FastTree 2--approximately maximum-likelihood trees for large alignments. PLoS One 5:e9490. doi: 10.1371/journal.pone.0009490, PMID: 20224823 PMC2835736

[ref44] Ren J. Ahlgren N. A. Lu Y. Y. Fuhrman J. A. Sun F. (2017). VirFinder: a novel k-mer based tool for identifying viral sequences from assembled metagenomic data. Microbiome 5:69. doi: 10.1186/s40168-017-0283-5, PMID: 28683828 PMC5501583

[ref45] Ren J. Song K. Deng C. Ahlgren N. A. Fuhrman J. A. Li Y.. (2020). Identifying viruses from metagenomic data using deep learning. Quant. Biol. 8, 64–77. doi: 10.1007/s40484-019-0187-4, PMID: 34084563 PMC8172088

[ref46] Rinke C. Rubino F. Messer L. F. Youssef N. Parks D. H. Chuvochina M.. (2019). A phylogenomic and ecological analysis of the globally abundant marine group II archaea (ca. Poseidoniales Ord. Nov.). ISME J. 13, 663–675. doi: 10.1038/s41396-018-0282-y, PMID: 30323263 PMC6461757

[ref47] Roux S. Enault F. Hurwitz B. L. Sullivan M. B. (2015). VirSorter: mining viral signal from microbial genomic data. PeerJ 3:e985. doi: 10.7717/peerj.985, PMID: 26038737 PMC4451026

[ref48] Santoro A. E. Richter R. A. Dupont C. L. (2019). Planktonic marine archaea. Annu. Rev. Mar. Sci. 11, 131–158. doi: 10.1146/annurev-marine-121916-063141 30212260

[ref49] Shannon P. Markiel A. Ozier O. Baliga N. S. Wang J. T. Ramage D.. (2003). Cytoscape: a software environment for integrated models of biomolecular interaction networks. Genome Res. 13, 2498–2504. doi: 10.1101/gr.1239303, PMID: 14597658 PMC403769

[ref50] Sun M. Zhan Y. Marsan D. Paez-Espino D. Cai L. Chen F. (2021). Uncultivated viral populations dominate estuarine Viromes on the spatiotemporal scale, Uncultivated viral populations dominate estuarine Viromes on the spatiotemporal scale. mSystems 6:6. doi: 10.1128/mSystems.01020-20, PMID: PMC8546989 33727395

[ref51] Tatusov R. L. Galperin M. Y. Natale D. A. Koonin E. V. (2000). The COG database: a tool for genome-scale analysis of protein functions and evolution. Nucleic Acids Res. 28, 33–36. doi: 10.1093/nar/28.1.33, PMID: 10592175 PMC102395

[ref52] Tully B. J. (2019). Metabolic diversity within the globally abundant marine group II Euryarchaea offers insight into ecological patterns. Nat. Commun. 10:271. doi: 10.1038/s41467-018-07840-4, PMID: 30655514 PMC6336850

[ref53] Vik D. R. Roux S. Brum J. R. Bolduc B. Emerson J. B. Padilla C. C.. (2017). Putative archaeal viruses from the mesopelagic ocean. PeerJ 5:e3428. doi: 10.7717/peerj.3428, PMID: 28630803 PMC5474096

[ref54] Von Meijenfeldt F. A. B. Arkhipova K. Cambuy D. D. Coutinho F. H. Dutilh B. E. (2019). Robust taxonomic classification of uncharted microbial sequences and bins with CAT and BAT. Genome Biol. 20:217. doi: 10.1186/s13059-019-1817-x, PMID: 31640809 PMC6805573

[ref55] Xie W. Luo H. Murugapiran S. K. Dodsworth J. A. Chen S. Sun Y.. (2018). Localized high abundance of marine group II archaea in the subtropical Pearl River estuary: implications for their niche adaptation. Environ. Microbiol. 20, 734–754. doi: 10.1111/1462-2920.14004, PMID: 29235710

[ref56] Xu B. Li F. Cai L. Zhang R. Fan L. Zhang C. (2022). A holistic genome dataset of bacteria, archaea and viruses of the Pearl River estuary. Sci Data 9:49. doi: 10.1038/s41597-022-01153-4, PMID: 35165305 PMC8844013

[ref57] Yamada K. D. Tomii K. Katoh K. (2016). Application of the MAFFT sequence alignment program to large data—reexamination of the usefulness of chained guide trees. Bioinformatics 32, 3246–3251. doi: 10.1093/bioinformatics/btw412%JBioinformatics 27378296 PMC5079479

[ref58] Zhang C. Du X. P. Zeng Y. H. Zhu J. M. Zhang S. J. Cai Z. H.. (2021). The communities and functional profiles of virioplankton along a salinity gradient in a subtropical estuary. Sci. Total Environ. 759:143499. doi: 10.1016/j.scitotenv.2020.143499, PMID: 33203567

[ref59] Zhang C. L. Xie W. Martin-Cuadrado A. B. Rodriguez-Valera F. (2015). Marine group II archaea, potentially important players in the global ocean carbon cycle. Front. Microbiol. 6:1108. doi: 10.3389/fmicb.2015.01108, PMID: 26528260 PMC4602124

[ref60] Zhou Y. Zhou L. Yan S. Chen L. Krupovic M. Wang Y. (2022). Diverse viruses of marine archaea discovered using metagenomics. Environ. Microbiol. 25, 367–382. doi: 10.1111/1462-2920.16287, PMID: 36385454

[ref61] Zimmerman A. E. Howard-Varona C. Needham D. M. John S. G. Worden A. Z. Sullivan M. B.. (2020). Metabolic and biogeochemical consequences of viral infection in aquatic ecosystems. Nat. Rev. Microbiol. 18, 21–34. doi: 10.1038/s41579-019-0270-x, PMID: 31690825

