# Supplementary figures and images for: Diversity, distribution, and functional potentials of magroviruses from marine and brackish waters
Source: Front Microbiol. 2023 Apr 21;14:1151034. doi: 10.3389/fmicb.2023.1151034 (PMC10160649; doi:10.3389/fmicb.2023.1151034)

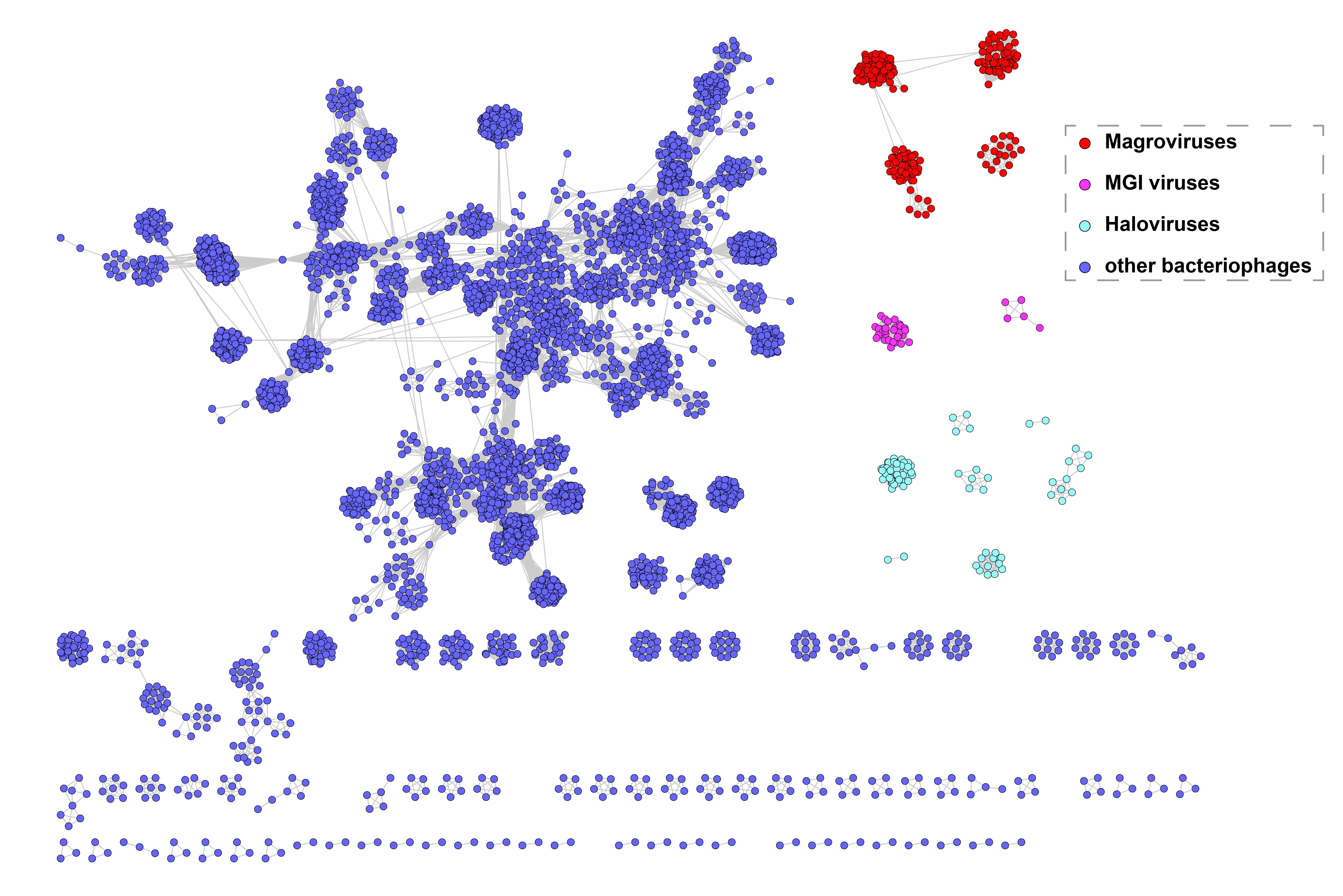

Supplement: Supplementary file 3 [file Image_1.JPEG]
